# Patterns of CSF Inflammatory Markers in Non-demented Older People: A Cluster Analysis

**DOI:** 10.3389/fnagi.2020.577685

**Published:** 2020-10-06

**Authors:** Yangdi Peng, Bin Chen, Lifen Chi, Qiang Zhou, Zhenjing Shi, The Alzheimer’s Disease Neuroimaging Initiative

**Affiliations:** AbbVie, Alzheimer’s Association; Alzheimer’s Drug Discovery Foundation; Araclon Biotech; BioClinica, Inc.; Biogen; Bristol-Myers Squibb Company; CereSpir, Inc.; Cogstate; Eisai Inc.; Elan Pharmaceuticals, Inc.; Eli Lilly and Company; EuroImmun; F. Hoffmann-La Roche Ltd.; and its affiliated company Genentech, Inc.; Fujirebio; GE Healthcare; IXICO Ltd.; Janssen Alzheimer Immunotherapy Research and Development, LLC.; Johnson and Johnson Pharmaceutical Research and Development LLC.; Lumosity; Lundbeck; Merck and Co., Inc.; Meso Scale Diagnostics, LLC.; NeuroRx Research; Neurotrack Technologies; Novartis Pharmaceuticals Corporation; Pfizer Inc.; Piramal Imaging; Servier; Takeda Pharmaceutical Company; and Transition Therapeutics; ^1^Department of Respiratory Medicine, Yongjia County Traditional Chinese Medicine Hospital, Wenzhou, China; ^2^Department of Neurology, Ruian People’s Hospital, The Third Affiliated Hospital of Wenzhou Medical University, Wenzhou, China; ^3^Department of Intervention, Ruian People’s Hospital, The Third Affiliated Hospital of Wenzhou Medical University, Wenzhou, China

**Keywords:** Alzheimer’s disease, inflammation, cytokines, tau, cluster analysis

## Abstract

**Objective:**

In this study, we aimed to examine if patterns of CSF inflammatory markers are correlated with global cognition, episodic memory, hippocampal volume, and CSF AD-related pathologies among non-demented older people.

**Methods:**

We included 217 non-demented older individuals, including 87 subjects with normal cognition (NC) and 130 subjects with mild cognitive impairment (MCI) from the Alzheimer’s Disease Neuroimaging Initiative (ADNI) study. Hierarchical cluster analysis including nine inflammatory markers in CSF [Tumor necrosis factor-α(TNF-α), TNF-R1, TNF-R2, transforming growth factor-β1 (TGF-β1), TGF-β2, TGF-β3, Interleukin-21 (IL-21), IL-6, and IL-7] was conducted.

**Results:**

We identified two clusters among non-demented older people based on nine inflammatory markers in CSF. Compared to the first cluster, the second cluster showed significantly higher levels of CSF inflammatory markers (TNF-R1, TNF-R2, TGF-β1, TGF-β3, and IL-6). Further, the second cluster was also associated with higher levels of *t*-tau and *p*-tau levels in CSF.

**Conclusion:**

We observed a subgroup of non-demented older people characterized by increased levels of inflammatory markers in CSF. Further, this subgroup showed higher levels of *t*-tau and *p*-tau levels in CSF.

## Introduction

Accumulating evidence has suggested that neuroinflammation plays a pivotal role in the pathogenesis of Alzheimer’s disease (AD) (Kinney et al., 2018; Guzman-Martinez et al., 2019; Wang et al., 2019). Several genetic studies have identified links between polymorphisms in genes associated with the immune system and the risk of developing AD (Calsolaro and Edison, 2016). In addition, previous studies have also observed changed levels of a variety of cytokines in patients with AD compared to subjects with normal cognition (NC) (Song et al., 2009; Craig-Schapiro et al., 2010; Réaux-Le Goazigo et al., 2013; Bettcher and Kramer, 2014). For instance, one study found that levels of soluble tumor necrosis factor receptor 1(TNFR1) and TNFR2 in CSF were positively associated with the BACE1 activity and tau levels in CSF among non-demented subjects, and that levels of soluble TNFR1 and TNFR2 in CSF were associated with the conversion from mild cognitive impairment (MCI) to AD dementia (Buchhave et al., 2010). Interleukin 6 (IL-6) was also found to be increased in the serum and CSF of patients with AD (Blum-Degen et al., 1995; Dursun et al., 2015). Additionally, in a 10-year longitudinal study, increased levels of peripheral IL-6 were found to be associated with steeper cognitive decline in late midlife (Singh-Manoux et al., 2014). However, given the fact that levels of these inflammatory markers are highly correlated with each other, it would be difficult to entangle the true relationships between inflammatory markers and AD-related outcomes. Alternatively, a data-driven approach could be used to identify subgroups based on several inflammatory markers. After that, differences in several AD-related outcomes can be examined between subgroups. This approach allows us to examine the relationship between patterns of inflammatory markers and AD-related outcomes.

In the present study, among non-demented subjects, cluster analysis was conducted to identify subgroups based on levels of nine inflammatory markers in CSF, including TNF-α, TNF-R1, TNF-R2, transforming growth factor-β1 (TGF-β1), TGF-β2, TGF-β3, IL-21, IL-6, and IL-7. Further, we compared several AD-related markers (global cognition, episodic memory, hippocampal volume and CSF AD pathologies) between subgroups.

## Materials and Methods

### Alzheimer’s Disease Neuroimaging Initiative

Data utilized in this work were extracted from the Alzheimer’s Disease Neuroimaging Initiative (ADNI) database^1^. The ADNI study was launched in 2003 as a public-private partnership, led by Principal Investigator Michael W. Weiner, MD. The ADNI study aims to examine whether clinical and neuropsychological assessment, serial magnetic resonance imaging (MRI), positron emission tomography (PET), and other biological markers can be combined to measure the progression of MCI and early AD. In the present study, we included 217 non-demented older individuals (130 males and 87 females), including 87 subjects with NC and 130 subjects with MCI. At each ADNI center across the United States and Canada, local institutional review board approved the ADNI study, and each subject provided written informed consent.

### Neuropsychological Assessments

The study participants underwent clinical and neuropsychological assessments in each ADNI center. In the present study, there were two cognitive outcomes: Mini-Mental State Examination (MMSE) (Folstein et al., 1975) and Rey Auditory Verbal Learning Test (RAVLT) (Schmidt, 1996) total learning score. These two assessments were used to examine the global cognition and verbal memory of study participants.

### Measurement of Hippocampal Volume

In an effort to examine neurodegeneration we utilized hippocampal volumes. The hippocampal volumes data were extracted from the ADNI file “ADNIMERGE.csv” (accessed August 2019). The neuroimaging techniques utilized by the ADNI have been detailed previously (Risacher and Saykin, 2013). Further information on neuroimaging methods could be found at the ADNI website^2^. In order to control the effect of sex difference in head size, adjusted hippocampal volumes were used in the present analysis. The formula is as following: Hippocampal/intracranial volumes ratio (HpVR) = hippocampal/intracranial volumes × 10^3^.

### Measurement of Aβ42, *t*-Tau, and *p*-Tau Levels in CSF

CSF AD pathologies (Aβ42, *t*-tau, and *p*-tau proteins) were analyzed by the multiplex xMAP Luminex platform and Innogenetics/Fujirebio AlzBio3 immunoassay reagents, details of which have been described previously (Shaw et al., 2009).

### Measurement of Levels of CSF Inflammatory Markers

The levels of CSF inflammatory markers (TNF-α, TNF-R1, TNF-R2, TGF-β1, TGF-β2, TGF-β3, IL-21, IL-6, and IL-7) were examined by William Hu and J. Christina Howell, Department of Neurology, Emory University. All these markers were examined by commercially available multiplex immunoassays (Millipore Sigma, Burlington, MA, United States). CSF inflammatory markers of the ADNI samples were examined in duplicate. Values are given in pg/ml.

### Statistical Analysis

**FIGURE 1:**
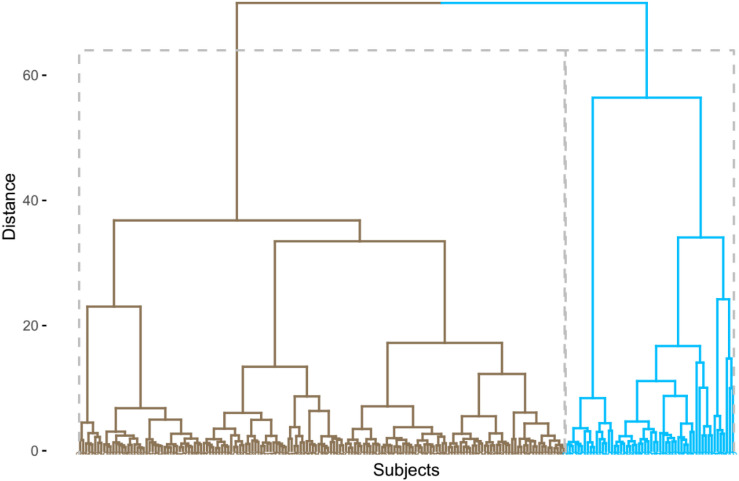
Dendrogram of non-demented older people based on nine CSF inflammatory proteins.

R statistical software (R Core Team, 2013) (version 3.6.0) was utilized to conduct an agglomerative hierarchical cluster analysis using nine CSF inflammatory proteins from 217 non-demented older people. Before clustering, these nine variables were standardized [(*x*-mean)/sd]. Cluster analysis could categorize participants so that participants are as similar to each other as possible within a cluster. Ward’s clustering linkage method (Ward, 1963) was utilized to combine participants while minimizing the error sum of squares. Each of the 217 non-demented older people was initially placed in their own cluster and then gradually combined with other participants. The results of the agglomerative hierarchical cluster analysis were displayed as a dendrogram (Figure 1). As a result, we identified two clusters among non-demented older people by cluster analysis. We further compared differences in demographics, clinical variables, and clustering variables between two clusters using *t*-tests and x^2^ tests. To examine whether two clusters demonstrate different levels of AD-related biomarkers (MMSE, RAVLT total learning score, HpVR, and CSF AD pathologies), *t*-tests were used. The significant variables (*p* < 0.1) identified in the *t*-tests were then entered in linear regression models with the adjustment of the potential effects of age, gender, educational years, clinical status, and APOE4 genotype. In addition, to examine whether gender modifies the relationship between cluster status and AD-related outcomes (MMSE, RAVLT total learning score, HpVR, and CSF AD pathologies), the clusters^∗^gender interaction term was added in our regression models.

## Results

### Cluster Analysis

In the present analysis, we identified two different clusters among non-demented older people (Figure 1). Compared to the first cluster (*n* = 161), the second cluster (*n* = 56) showed significantly higher levels of CSF TNF-R1, TNF-R2, TGF-β1, TGF-β3, and IL-6 (Table 1). However, no significant differences in CSF TNF-α, TGF-β2, IL-21, or IL-7 levels were found between two clusters (Table 1).

**TABLE 1:** Demographical and clinical variables between two clusters.

Characteristics	Cluster 1 (*n* = 161)	Cluster 2 (*n* = 56)	*P*-values
Age, years	73.9 ± 6.82	77.8 ± 6.58	<0.001
Education, years	15.7 ± 2.8	15.9 ± 3.47	0.66
Female gender, *n* (%)	72 (44.7)	15 (26.8)	0.018
APOE4 carriers, *n* (%)	70 (43.5)	22 (39.3)	0.58
MCI diagnosis, *n* (%)	98 (60.9)	32 (57.1)	0.62
MMSE scores	27.8 ± 1.86	27.7 ± 1.82	0.81
RAVLT total learning score	35.5 ± 10.9	34.1 ± 10.5	0.38
HpVR	4.39 ± 0.75	4.24 ± 0.66	0.22
CSF Aβ42 levels, pg/ml	184 ± 55.7	189 ± 58.4	0.56
CSF *t*-tau levels, pg/ml	81.7 ± 45.4	103 ± 67.7	0.03
CSF *p*-tau levels, pg/ml	29 ± 15.2	35.2 ± 21.5	0.050
**CSF inflammatory markers, pg/ml**			
TNF-α	1.62 ± 0.427	2.74 ± 3.48	0.02
TNF-R1	816 ± 166	1038 ± 315	<0.001
TNF-R2	961 ± 195	1372 ± 725	<0.001
TGF-β1	93.7 ± 26.2	144 ± 51	<0.001
TGF-β2	162 ± 52.7	162 ± 38.4	0.995
TGF-β3	2.81 ± 0.517	32.3 ± 45.5	<0.001
IL-21	11.1 ± 11.6	13.2 ± 15.9	0.37
IL-6	4.19 ± 2.11	8.4 ± 9.37	0.002
IL-7	1.11 ± 0.746	1.76 ± 3.48	0.17

*MCI, mild cognitive impairment; MMSE, mini-mental state examination; RAVLT, Rey Auditory Verbal Learning Test; HpVR, hippocampal/intracranial volumes ratio; TNF- α, Tumor Necrosis Factor- α; TNF-R1, Tumor Necrosis Factor Receptor 1; TNF-R2, Tumor Necrosis Factor Receptor 2; TGF-β1, Transforming Growth Factor-β1; TGF-β2, Transforming Growth Factor-β2; TGF-β3, Transforming Growth Factor-β3; IL, Interleukin.*

### Demographics and Clinical Variables Between Clusters

As shown in Table 1, the first cluster were younger and more likely to be women than the second cluster. However, no differences in educational years, percentage of APOE4 carriers, or percentage of MCI individuals were found between two clusters (all *p* > 0.05).

### AD-Related Markers Between Two Clusters

**FIGURE 2:**
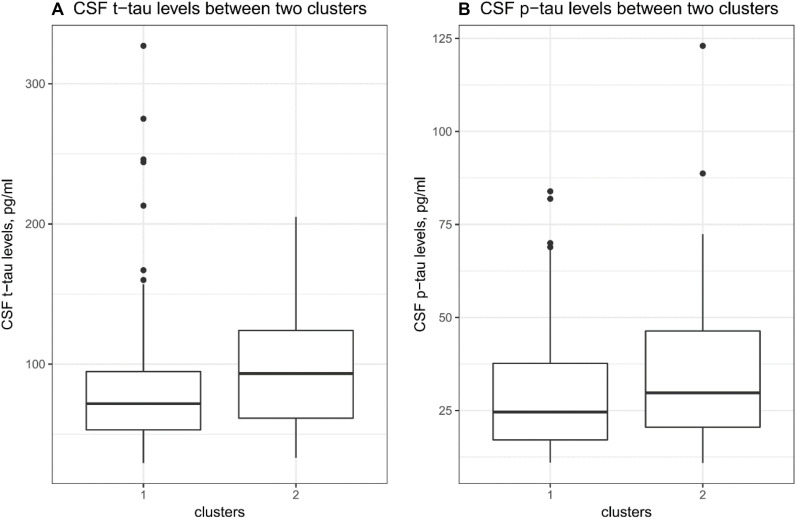
CSF tau pathologies between clusters. **(A)** There was a significant difference in CSF t-tau levels between two clusters (*p* = 0.03). **(B)** There was a marginally significant difference in CSF *p*-tau levels between two clusters (*p* = 0.05).

To examine whether two clusters demonstrate different levels of AD-related markers, several t tests were performed. As shown in Table 1 and Figure 2A, compared to the first cluster, the second cluster showed significantly higher levels of CSF *t*-tau (*p* = 0.03). Further, the differences in CSF *p*-tau levels between two clusters were marginally significant (*p* = 0.05; Table 1 and Figure 2B). However, no differences in MMSE, RAVLT total learning score, HpVR or CSF Aβ42 were found between two clusters (Table 1).

To further investigate the relationship between cluster status and CSF tau pathologies, several linear regression models were performed with the adjustment of several potential covariates. As shown in Table 2, compared to the first cluster, the second cluster showed significantly higher levels of CSF tau pathologies (*t*-tau and *p*-tau) after controlling for other potential covariates.

**TABLE 2:** Summary of linear regression models.

Independent variables	*t*-tau		*p*-tau	
	B (SE)	*P*-values	B (SE)	*P*-values
Cluster 2 vs Cluster 1	16.5 (6.8)	0.015	6.5 (2.6)	0.012
Age	0.58 (0.42)	0.17	0.27 (0.16)	0.1
Female gender	9.2 (6.1)	0.13	4.2 (2.3)	0.07
Educational years	−0.22 (0.97)	0.82	0.3 (0.37)	0.41
APOE4 carriers	20.8 (6)	<0.001	9.9 (2.3)	<0.001
MCI diagnosis	27.2 (6)	<0.001	6.4 (2.3)	0.006

*MCI, mild cognitive impairment. B represents unstandardized β; SE represents standard error.*

## Supplementary Analysis

In addition, we further tested whether gender modifies the relationship between cluster status between each AD-related outcome (MMSE, RAVLT total learning score, HpVR, and CSF AD pathologies) in our regression models. The cluster status^∗^gender interaction term was added in our regression models with adjustment of other covariates (age, education, APOE4 genotype and clinical diagnosis). However, this interaction term was not significant for any AD-related outcome (all *p* > 0.05, MMSE, RAVLT total scores, HpVR or CSF AD biomarkers). This indicated that gender did not modify the relationship between cluster status and AD-related outcomes.

## Discussion

In the present study, we observed a subgroup of non-demented older people characterized by increased levels of inflammatory markers in CSF (TNF-R1, TNF-R2, TGF-β1, TGF-β3, and IL-6). To our knowledge, this is the first study to show a subgroup of non-demented older people based on levels of nine inflammatory markers in CSF using cluster analysis. In addition, this cluster showed higher levels of *t*-tau and *p*-tau levels in CSF.

Some data-driven methods, such as cluster analysis, can categorize participants into several subgroups based on heterogeneity within a group of biomarkers. Previously, several studies have been conducted using this approach among healthy controls and patients with cognitive impairment (Iqbal et al., 2005; van der Vlies et al., 2009; Nettiksimmons et al., 2010, 2013, 2014; Wallin et al., 2010; Escudero et al., 2011; Skillbäck et al., 2013; Noh et al., 2014; Racine et al., 2016). However, no previous studies using cluster analysis have attempted to examine levels of these inflammatory markers in CSF to identify potential subgroups among non-demented older people. In this study, we observed two subgroups of non-demented older people. Cluster 1 was characterized by low levels of several inflammatory markers in CSF (TNF-R1, TNF-R2, TGF-β1, TGF-β3, and IL-6) while Cluster 2 by increased levels of these markers in CSF (Table 1). However, no significant differences in CSF TNF-α, TGF-β2, IL-21, or IL-7 levels were found between two clusters (Table 1 and Figure 1). Compared to participants in Cluster 1, those in Cluster 2 were older and had a higher percentage of men (Table 1).

Further, we found that the second cluster demonstrated higher levels of *t*-tau and *p*-tau in CSF compared to the first cluster, indicating that neuroinflammation may play a crucial role in the pathogenesis of AD. A recent meta-analysis involving 170 studies showed that levels of multiple inflammatory markers were substantially altered in comparison between AD, MCI and healthy control, also suggesting an important role of inflammation in the development of AD (Shen et al., 2019). In addition, alterations in several inflammatory markers, such as TNF-α-related cytokines levels in plasma, have been reported to be associated with cognitive decline in AD (Bettcher and Kramer, 2014; Hye et al., 2014). In line with our findings, a previous study also showed that six inflammatory markers were positively correlated with levels of *t*-tau and *p*-tau in CSF among subjects with NC and patients with cognitive impairment (Popp et al., 2017). In addition, Buchhave and colleagues found that levels of soluble TNFRs in CSF were positively correlated with CSF tau levels in healthy controls and MCI subjects (Buchhave et al., 2010). A previous animal study suggested that induction of inflammation could contribute to tau hyperphosphorylation (Kitazawa et al., 2005). On the contrary, in tau transgenic model, the early immunosuppression could lead to the reduction of tau pathology and increased lifespan (Yoshiyama et al., 2007). Taken together, previous findings and ours suggested that inflammation may play a critical role in the development of AD.

However, we did not find a significant difference in CSF Aβ42 between two clusters. This finding is consistent with a previous study showing that inflammatory markers were not correlated with CSF Aβ42 (Popp et al., 2017). In AD models, microglial inhibition can decrease neuronal loss, while it does not affect levels of amyloid-β and plague load (Spangenberg et al., 2016), indicating that the production of amyloid in AD may rely on other mechanisms.

Several limitations should be noted. First, given the cross-sectional nature of this study, we cannot clarify the temporal relationship between clusters and tau pathology in CSF among non-demented older people. Therefore, further longitudinal studies would be important to examine the association between inflammatory patterns and change in tau pathology in CSF over time. Second, given that participants of the ADNI study are dominantly white and well-educated, this may limit our findings to be generalized to other populations. Therefore, population-based studies should be conducted. Third, it would be interesting to examine the relationship between cluster status and the conversion rate from control to MCI or from MCI to AD dementia, future longitudinal studies should be conducted to examine these research questions.

In conclusion, we observed a subgroup of non-demented older people characterized by increased levels of inflammatory markers in CSF (TNF-R1, TNF-R2, TGF-β1, TGF-β3, and IL-6). Further, this subgroup showed higher levels of *t*-tau and *p*-tau levels in CSF.

## Data Availability Statement

The datasets presented in this article are not readily available because we downloaded data from the ADNI study, which is open to the public (adni.loni.usc.edu). Requests to access the datasets should be directed to adni.loni.usc.edu.

## Ethics Statement

The studies involving human participants were reviewed and approved by at each ADNI center across the United States and Canada, local institutional review board approved the ADNI study, and each subject provided written informed consent. The patients/participants provided their written informed consent to participate in this study.

## Author Contributions

ZS and YP conceived and designed the study. YP, BC, LC, and QZ performed the research and analyzed the data. YP wrote the manuscript. All authors approved the final version of the manuscript.

## Conflict of Interest

The authors declare that the research was conducted in the absence of any commercial or financial relationships that could be construed as a potential conflict of interest.

## Footnotes

1 adni.loni.usc.edu

2 www.adni-info.org/methods
